# Editorial: Soil-transmitted helminth infections from a One Health perspective

**DOI:** 10.3389/fmed.2023.1167812

**Published:** 2023-04-11

**Authors:** Joel Henrique Ellwanger, Serena Cavallero

**Affiliations:** ^1^Laboratory of Immunobiology and Immunogenetics, Postgraduate Program in Genetics and Molecular Biology (PPGBM), Department of Genetics, Universidade Federal do Rio Grande do Sul (UFRGS), Porto Alegre, Brazil; ^2^Department of Public Health and Infectious Diseases, Sapienza University of Rome, Rome, Italy

**Keywords:** infection, hookworm, One Health, parasites, public health, roundworm, soil-transmitted helminths, whipworm

## 1. The One Health perspective

One Health is a multidisciplinary approach aimed at protecting and promoting the health of humans, animals, and ecosystems (1). In a public health context, One Health is particularly applied to investigate, understand, control, and prevent infectious and parasitic diseases, with particular attention to zoonoses. One Health highlights that the health of (I) human societies, (II) domestic and wild animals, and (III) the environment is strongly connected and, therefore, public health issues must be addressed by considering such connections (1). Several examples highlight these aspects in the context of infectious and parasitic diseases. Anthropogenic activities that are detrimental to the environment and animals facilitate the transmission of several zoonotic parasites (2). Pervasive human interaction with wild species (e.g., in wet markets) contributed to the emergence of SARS-CoV-2 in the human population and the COVID-19 pandemic (3). Deforestation of tropical forests and habitat fragmentation facilitate the dissemination of several viral, bacterial, and parasitic diseases. By contrast, the preservation of biodiversity and the environment is a protective factor against emerging infectious diseases (4). Currently, there is a growing acceptance of the One Health need to address parasitic diseases in the next decades in a changing, conflicted, and resource-limited world (5, 6).

## 2. Soil-transmitted helminths from a One Health perspective

Soil-transmitted helminths (STHs) are a group of parasites that mainly affect populations in places with sanitation problems as STH transmission particularly occurs through contact with soil contaminated with parasitic eggs or larvae (7, 8). It is estimated that 1.5 billion individuals are infected with STHs worldwide and they are included in the list of neglected tropical diseases by the WHO (8). STH infections cause several health problems, such as malnutrition, iron deficiency, and child developmental deficits (7, 8). Among STHs, roundworms (*Ascaris lumbricoides*), whipworms (*Trichuris trichiura*), hookworms (*Necator americanus* and *Ancylostoma duodenale*), and *Strongyloides stercoralis* are of great public health importance for some populations. Zoonotic STHs (parasites transmitted from animals to humans), including *Strongyloides fuelleborni, Toxocara canis, Toxocara cati, Ancylostoma ceylanicum*, and *Ancylostoma brazilienese*, also cause concern in several countries (7).

**Figure 1:**
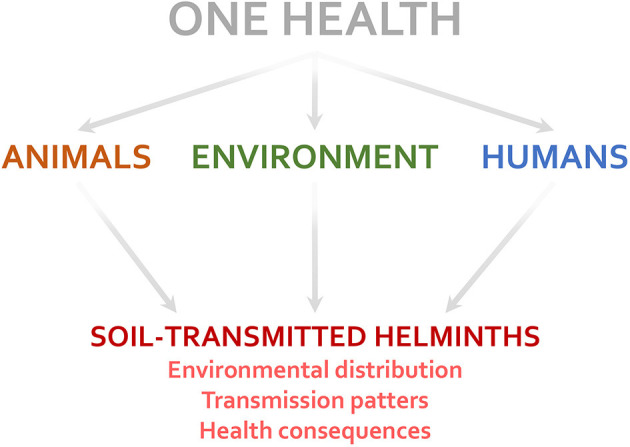
Factors related to the One Health triad (humans, animals, and the environment) influence several aspects of soil-transmitted helminth infections.

Animal, environmental, and human factors may affect multiple aspects associated with STH infections (Figure 1). Vertebrate animals, such as dogs and cats, influence the risk of zoonotic STH infections (by *T. canis, T. cati*, etc.) (9). Invertebrate animals can act as mechanical carriers of parasite eggs between different places (10). The lack of environmental sanitation and access to clean water facilitate the contamination of soil and foods with eggs and larvae of STHs, increasing infection risk (8). Human immunological, nutritional, and genetic factors may influence both susceptibility to and consequences of STH infection in the host (e.g., parasite load, affecting the number of eggs excreted in the feces) (7, 11, 12). Socio-economic and cultural factors (e.g., open defecation, walking barefoot, and food habits) also modify the STH infection transmission risk (12, 13). Further discussion about the importance of addressing parasitic diseases under the One Health perspective can be found elsewhere (14, 15).

## 3. New findings

The articles published in this Research Topic advanced the knowledge of STHs, with contributions from different perspectives. In a study performed in Slovakia, Ihnacik et al. investigated STHs from the One Health perspective, evaluating human, animal, and environmental (soil) samples. The study focused on the Roma community, a socially vulnerable group of people. In human samples, *A. lumbricoides* and *T. trichiura* were the most prevalent parasites. Of note, people belonging to the Roma minority had a higher infection rate (odds ratio: 37.12) than non-Roma inhabitants, highlighting the importance of social issues as determinants of STH infections. Considering the parasitological evaluation of the environment, 26.26% of soil samples were positive for STH eggs. Regarding domestic animals, the authors observed STH eggs in 43.55% of the analyzed dog fecal samples. Finally, Ihnacik et al. reported that climatic factors, ethnicity, and water sanitation and hygiene (WASH) issues are crucial factors influencing the distribution and transmission of STHs in the studied population, suggesting the need for a One Health approach to control STH infection in Slovakia. Of note, other studies performed in different countries supported the role of climatic factors (16, 17), ethnicity (18, 19), and WASH (20, 21) as determinants of STH distribution and transmission.

Helminth infections alter immune responses in different ways, often influencing the susceptibility and outcome of non-parasitic diseases. In this context, Bazargan et al. performed a case-control study and scoping review of the literature to investigate the influence of *Toxocara* infection on allergic asthma manifestations in humans. For the case-control study, data from 124 healthy individuals and 124 patients with asthma from Iran were evaluated. The authors observed a significant association between asthma severity and age in *Toxocara*-seropositive individuals. However, no significant association between asthma and *Toxocara* seropositivity was found. Bazargan et al. suggest the need for further studies investigating the potential influence of *Toxocara* infection on asthma susceptibility in humans. Case-control, cross-sectional, and cohort studies are a few methodological approaches that can be used for such an investigation.

Malaria and helminth co-infection is a major issue in some African countries. Afolabi et al. performed two surveys among children living in urban and rural settings in Senegal to determine the prevalence of malaria-helminth co-infection. There was a 2.2% prevalence of polyparasitism with *Plasmodium falciparum*, STHs, *Schistosoma haematobium*, and *Schistosoma mansoni* among children in the two study sites. The prevalence of *P. falciparum*-*S. haematobium* and *P. falciparum*-*S. mansoni* co-infections was 1.1% and 0.7%, respectively. These co-infection rates were considered low, a result potentially linked to the sustained effective control measures for parasitic infections.

Finally, Walker et al. evaluated the effectiveness of One Health interventions against the zoonotic hookworm *Ancylostoma ceylanicum*, a parasite of dogs but also commonly reported in people from Southeast Asia and the Pacific. The authors modeled the effect of mass drug administration (MDA) on infection control, showing that targeting both dogs and humans could suppress prevalence in humans to ≤1% by the end of 2030, with a 25–50% coverage of the animal reservoir. The study also suggested that the disease transmission cycle could be completely interrupted with an increase in treatment coverage. In brief, this study evidenced the importance of controlling zoonotic STH infections from a One Health perspective.

## 4. Conclusion

STH infections should be studied and managed with the One Health perspective in mind, as strongly suggested by the studies included in this Research Topic. This approach is increasingly important in a world with huge environmental and social challenges.

## Author contributions

JE wrote the first draft of the manuscript and prepared Figure 1. SC complemented and edited the text. All authors contributed to the article and approved the submitted version.
